# Point Mutations in GLI3 Lead to Misregulation of its Subcellular Localization

**DOI:** 10.1371/journal.pone.0007471

**Published:** 2009-10-15

**Authors:** Sybille Krauß, Joyce So, Melanie Hambrock, Andrea Köhler, Melanie Kunath, Constance Scharff, Martina Wessling, Karl-Heinz Grzeschik, Rainer Schneider, Susann Schweiger

**Affiliations:** 1 Charité University Hospital, Department of Dermatology, Berlin, Germany; 2 Max-Planck Institute for Molecular Genetics, Department of Human Molecular Genetics (Ropers), Berlin, Germany; 3 Institute of Biochemistry and Center for Molecular Biosciences Innsbruck (CMBI), Innsbruck, Austria; 4 Center for Human Genetics, Phillipps University, Marburg, Germany; 5 Ninewells Hospital, Department of Neuroscience and Pathology, Dundee, United Kingdom; Universität Heidelberg, Germany

## Abstract

**Background:**

Mutations in the transcription factor GLI3, a downstream target of Sonic Hedgehog (SHH) signaling, are responsible for the development of malformation syndromes such as Greig-cephalopolysyndactyly-syndrome (GCPS), or Pallister-Hall-syndrome (PHS). Mutations that lead to loss of function of the protein and to haploinsufficiency cause GCPS, while truncating mutations that result in constitutive repressor function of GLI3 lead to PHS. As an exception, some point mutations in the C-terminal part of GLI3 observed in GCPS patients have so far not been linked to loss of function. We have shown recently that protein phosphatase 2A (PP2A) regulates the nuclear localization and transcriptional activity a of GLI3 function.

**Principal Findings:**

We have shown recently that protein phosphatase 2A (PP2A) and the ubiquitin ligase MID1 regulate the nuclear localization and transcriptional activity of GLI3. Here we show mapping of the functional interaction between the MID1-α4-PP2A complex and GLI3 to a region between amino acid 568-1100 of GLI3. Furthermore we demonstrate that GCPS-associated point mutations, that are located in that region, lead to misregulation of the nuclear GLI3-localization and transcriptional activity. GLI3 phosphorylation itself however appears independent of its localization and remains untouched by either of the point mutations and by PP2A-activity, which suggests involvement of an as yet unknown GLI3 interaction partner, the phosphorylation status of which is regulated by PP2A activity, in the control of GLI3 subcellular localization and activity.

**Conclusions:**

The present findings provide an explanation for the pathogenesis of GCPS in patients carrying C-terminal point mutations, and close the gap in our understanding of how GLI3-genotypes give rise to particular phenotypes. Furthermore, they provide a molecular explanation for the phenotypic overlap between Opitz syndrome patients with dysregulated PP2A-activity and syndromes caused by GLI3-mutations.

## Introduction

GLI3 and two other GLI-proteins, GLI1 and GLI2, are mammalian homologues of the *Drosophila* protein Cubitus interruptus (Ci), which is a transcriptional effector of Hedgehog (Hh) signaling. Ci was shown to be part of a microtubule-associated protein complex that also contains Fused (Fu), Suppressor of Fused (SuFu) and Costal2 (Cos2). In absence of Hh signaling, Ci gets phosphorylated by PKA and is subsequently cleaved by the proteasome. The N-terminal cleavage product enters the nucleus and acts as a transcriptional repressor of target genes. In the presence of the Hh signal, this cleavage is blocked and Ci matures into a transcriptional activator [1]–[4]. Similar to its *Drosophila* homologue, GLI3 undergoes PKA-dependent cleavage and can act as either a transcriptional activator or repressor of target genes in the mammalian Sonic Hedgehog (SHH) pathway [5]–[10].

Among other genetic syndromes, mutations in GLI3 are found in patients with Greig-cephalopolysyndactyly-syndrome (GCPS [MIM175700]) [11], [12], Pallister-Hall syndrome (PHS [MIM146510]) [13], [14] and - in one patient - acrocallosal syndrome [MIM200990] [15]. Several studies have discussed putative genotype-phenotype correlations to explain how mutations in the same gene could cause clinically distinct syndromes. While mutations leading to C-terminally truncated GLI3, which functions as a constitutive repressor, are responsible for the development of PHS [6], [16], N-terminal loss-of-function mutations lead to haploinsufficiency and cause GCPS [5], [12], [17]. However, in some cases of GCPS missense mutations in the C-terminal part of GLI3 were observed that could not be correlated with loss of function of the protein and therefore did not seem to fit into this genotype-phenotype correlation [11], [13].

Using several cancer cell lines, we have recently shown that protein phosphatase 2A (PP2A) regulates the subcellular localization and transcriptional activity of GLI3 [18]. An increase in PP2A activity results in the cytosolic retention and decreased transcriptional activity of GLI3, while inhibition of PP2A leads to nuclear accumulation and increased transcriptional activity. We have further seen that this mechanism is critically influenced by the MID1-α4-ubiquitin-ligase complex that targets microtubule-associated PP2Ac (catalytic subunit of PP2A) to ubiquitin-specific modification and degradation [19]. Interestingly, the three GLI3-related conditions GCPS, PHS and acrocallosal syndrome show phenotypic overlap with Opitz BBB/G syndrome (OS [MIM300000 and 145410]), a midline malformation syndrome caused by loss-of-function mutations of MID1 [20]. While patients with GCPS share facial features such as hypertelorism and broad nasal bridge with OS patients, PHS patients can present with cleft lip/palate and laryngotracheal and anal malformations, which can also be found in OS patients. Patients with acrocallosal syndrome however, similar to OS patients, can show a full spectrum of midline malformations, including hypertelorism, broad nasal bridge, cleft lip/palate, congenital heart defects, imperforate anus and hypospadias.

In this study, we have narrowed down the protein region of GLI3 that is necessary for its functional interaction with the MID1-α4-PP2A complex. Furthermore, we show that missense mutations in this region, as observed in GCPS and acrocallosal syndrome patients, lead to changes in the subcellular localization of the GLI3 protein and interfere with its regulation by the MID1-α4-PP2A complex. Our data give insight into a molecular cross-talk between the MID1-α4-PP2A complex and GLI3, paving the way to a molecular explanation for the loss-of-function phenotype in GCPS patients with C-terminal missense mutations. In addition, they provide evidence for a shared molecular mechanism underlying several malformation syndromes with significant phenotypic overlap.

## Materials and Methods

### Constructs

All expression-vector-constructs are listed in table 1. Sequences of all mutants have been confirmed by direct sequencing.

**Table 1:** Vector-information and cloning strategies of all cDNA-expression constructs used in this study.

clone	cDNA coding for/insert	vector	Enzymes used for cloning/strategy/publication
fpc18-1549	GLI3 AA 18-1549	pEGFP-C1 (Clontech)	*EcoRI-SmaI*
fpn1-1522	GLI3 AA 1-1522	pEGFP-N1 (Clontech)	*Bsp120I-SacI*
fpn1-396	GLI3 AA 1-396	pEGFP-N2 (Clontech)	*BamHI*
fpn1-1100	GLI3 AA 1-1100	pEGFP-N3 (Clontech)	*SacI-SmaI*
fpn824-1100	GLI3 AA 824-1100		fpn 1-1100 digested with *EcoRI-Bsp120I*, removal of a 2412 bp fragment (GLI3 aa 1-823), religation
fpc18-667	GLI3 AA 18-667	pEGFP-C1 (Clontech)	*EcoRI-Bsp120I*
fpc18-828	GLI3 AA 18-828	pEGFP-C1 (Clontech)	*EcoRI-SmaI*
fpc18-1100	GLI3 AA 18-1100	pEGFP-C1 (Clontech)	*EcoRI-SmaI*
fpc568-1549	GLI3 AA 568-1549		fpc 18-1549 digested with *BglII*, removal of a 1756bp fragment (GLI3 aa 18-585), religation
200-1580	GLI3 AA 200-1580	pEGFP-C1 (Clontech)	*SacI-SalI*
400-1580	GLI3 AA 400-1580	pEGFP-C1 (Clontech)	*SacI-SalI*
8x3 GLI-BS Luc	8 directly repeated copies of a GLI-binding site (GAACACCCA)	pδ51LucII [44]	[*45*]
8x3 MutGLI-BS Luc	8 directly repeated copies of a mutant GLI-binding site (GAAgtgggA)	pδ51LucII [44]	[*45*]
pRL	Renilla luciferase	pRL (Promega)	
A934P	GLI3 AA 18-1549 mutation A934P	pEGFP-C1 (Clontech)	Produced by in-vitro mutagenesis on fpc18-1549
I808M	GLI3 AA 18-1549 mutation I808M	pEGFP-C1 (Clontech)	Produced by in-vitro mutagenesis on fpc18-1549
P707S	GLI3 AA 18-1549 mutation P707S	pEGFP-C1 (Clontech)	Produced by in-vitro mutagenesis on fpc18-1549
MID1-myc	MID1 complete cDNA	pCMVTag3A (Stratagene)	*HinDIII-EcoRI*
GLI3-Flag	GLI3 AA 18-1580	pCMVTag2A (Stratagene)	*EcoRI*

### Immunofluorescence

For immunofluorescence experiments, HeLa or U373MG cells were plated on coverslips (in 6-well plates) at a density of 1×10^5^ cells per well one day before transfection. Cells were transfected with the respective plasmids using Polyfect transfection reagent (Qiagen) according to the manufacturer's instructions. Twenty-four hours after transfection, cells were fixed with 3.7% paraformaldehyde in PEM buffer (10×PEM contains 1 M Pipes, 0.05 M EGTA, 0.02 M MgCl_2_, pH 7.0). The GFP-GLI3 signal distribution in individual cells occurred in three patterns: exclusively nuclear fluorescence, even staining throughout the cytosol and nucleus, or predominantly cytosolic fluorescence. 100 transfected cells per coverslip were counted and classified in one of the three groups. Each experiment was repeated in triplicates. Data shown represent mean ± s.d. scored per group from 3 independent experiments of 100 cells each. Statistical significance was evaluated using T-Test (two-tailed, homoscedastic). Microscopists were blinded to the nature of the constructs.

### Transfection, cell fractionation and Western blotting

1×10^5^ HeLa cells per well of a 6-well plate were seeded 24 hours prior transfection. Transfections with the respective plasmids were performed using Polyfect transfection reagent (Qiagen) according to the manufacturer's instructions. For cell fractionation, 48 hours after transfection cells were resuspended in fractionation buffer (40 µg/ml digitonin, 50 mM Tris-HCl, pH 7.5, 100 mM NaCl, 2.5 mM MgCl_2_) and incubated for 10 min at room temperature. Nuclear and cytosolic fractions were separated by centrifugation (1000 x g, 10 min, 4°C) and mixed with 2× Magic Mix (48% urea, 15 mM Tris-HCl, 8.7% glycerol, 1% SDS, 0.004% bromphenol blue, 143 mM β-mercaptoethanol). For analysis of total cell lysates cells were resuspended in Magic Mix 48 hours after transfection. Samples were denatured at 95°C, separated on SDS-Gels and blotted on PVDF membranes (Roche).

### Antibodies

All antibodies used in this study are listed in table 2.

**Table 2:** Antibodies used in this study.

antibody	company	Catalogue number	publication
α4			[19]
Actin	SIGMA	A2066	
GAPDH	Abcam	Ab9485	
Flag	Stratagene	200471	
Myc	Clontech	3800-1	
GFP	Abcam	Ab6673	
GFP	Roche	1814416	
Cy3-anti-mouse	Dianova	715-166-151	
HRP-anti-mouse	Dianova	115-035-003	
HRP-anti-rabbit	Amersham	NA 9340	
HRP-anti-goat	Santa Cruz	Sc-2020	
phosphoserine sampler kit	Biomol	54694	
phosphothreonine sampler kit	Biomol	54695	

### RNAi

Twenty-four hours before transfection, HeLa cells were seeded into 6-well plates at a density of 1×10^5^ per well. Cells were transfected with 1.3 µg of synthetic siRNA per well using Oligofectamine (Invitrogen) according to the manufacturer's instructions. Sequences of siRNA's are listed (Table S1a).

### Real-time PCR

Twenty-four hours before treatment, HeLa cells were seeded at a density of 1×10^5^ cells per well of a 6-well plate. Cells were transfected with siRNA's as described. Total RNA was isolated using an RNeasy Mini Kit (Qiagen) following the manufacturer's instructions. cDNA was synthesized using the TaqMan reverse transcription reagents kit (Applied Biosystems) and real-time PCR was carried out using the SYBRGreen PCR master mix (Applied Biosystems) according to the manufacturer's instructions with an ABI 7900HT cycler under the following conditions: 50°C for 2 min; 95°C for 10 min; 95°C for 15 sec, 60°C for 1 min for 40 cycles; and 95°C for 15 min, 60°C for 15 min, 95°C for 15 min for the dissociation stage. Sequences of primers are listed (Table S1b).

### Luciferase reporter assay

4×10^4^ HeLa cells per well of a 12-well plate were seeded 24 hours prior transfection. Reporter plasmids containing eight either wildtype (wt) or mutant GLI binding sites linked to firefly luciferase (1.5 µg DNA per well) were co-transfected with GLI3-wt or mutant constructs (1 µg DNA per well) and renilla luciferase (1 ng DNA per well) for normalization. Transfections with the respective plasmids were performed using JetPEI (Polyplus Transfections) according to the manufacturer's instructions. The reporter assays were carried out using the Dual-Luciferase Reporter 1000 Assay System (Promega) following the manufacturer's instructions. Luciferase intensities were measured in a Centro Luminometer LB 960 (Berthold).

### 3D microscopy

For 3D microscopy experiments, HeLa cells were plated on coverslips (in 6-well plates) at a density of 1×10^5^ cells per well one day prior transfection. Cells were transfected with the respective plasmids using Polyfect transfection reagent (Qiagen) according to the manufacturer's instructions. 24 hours after transfection cells were fixed with 3.7% paraformaldehyde in PEM (10×PEM contains 1 M Pipes, 0.05 M EGTA, 0.02 M MgCl_2_, pH 7.0). Afterwards cells were permeabilized with 0.2% TritonX100 and blocked with 1% BSA. Antibody incubations were carried out following the manufacturer's instructions. 3D colocalization studies were performed using an epifluorescence microscope (Axioskop; Carl Zeiss MicroImaging, Inc.) equipped with a 100x-plan-neofluar oil-immersion lens (NA 1.35; Carl Zeiss MicroImaging, Inc.) attached to a PIFOC z-SCAN (Physik Instrumente), and a 12-bit CCD digital camera (SensiCam; PCO) controlled by TILLvisION v4.0 software. Fluorochromes were excited using a polychrome IV monochromator (T.I.L.L. Photonics) in combination with a quadruple band pass beam splitter and barrier filter (Chroma Technology Corp.) allowing subsequent recording of blue (DAPI), red (Cy3), green (GFP) fluorescence for the same focal plane. Spatial relationship of protein signals was determined by interactively scanning through the image stacks. Deconvolution was performed using the cMLE algorithm of Huygens 2.1.4-essential (Scientific Volume Imaging) running under Windows NT. Rendering of 3D data was done using the Surpass module of Imaris 3.3.2 (Bitplane).

Confocal microscopy localization studies were performed using an inverse laser scanning microscope (LSM510 META, Axiovert 200M) equipped with a 63x-plan-neofluar oil-immersion lens (Plan Apochromat 63x/1.4oil) controlled by LSM510 4.0 SP2 (ZEISS) software. Fluorescence signals were captured with the filters BP 505-530 for EGFP and BP 420-480 for DAPI. Images were analyzed using the LSM Image Browser Version 4.2.0.121 (ZEISS).

### 2D-Gel electrophoresis

2D-Gel electrophoresis was carried out as described elsewhere [21]. The following modifications were made to the protocol:

1.6×10^6^ HeLa cells were seeded in 150 cm^2^ flasks 1 day prior transfection. Cells were transfected using Polyfect (Qiagen) and fractionated 48 hours after transfection in fractionation buffer [10 mM Tris pH 7.5, 2.5 mM MgCl_2_, 10 mM DTT, PhosStop (phosphatase inhibitor cocktail; Roche), complete (protease inhibitor cocktail; Roche), 80 µg/ml Digitonin] as described (see Western blot). Samples were treated with 500 U/ml benzonase (Merck). Afterwards 4% Triton X-100, 9 M urea, and 0.4% mixed ampholytes (composition see [21]) were added. IEF gels were prepared as described [21] and run in a Mini Tube Gel Cell for 2-D Electrophoresis (Bio-Rad) under the following conditions: 1 h 100 V; 1 h 200 V; 15 h 400 V; 1 h 600 V; 10 min 800 V; 5 min 1000 V. IEF gels were equilibrated (as described [21]) and transferred to SDS gels which were run in a Mini-PROTEAN Tetra Electrophoresis System (Bio-Rad) at 200 V. Afterwards SDS gels were subjected to Western blotting.

### Phos-Tag

For mobility shift detection of phosphorylated proteins Phos-tag technology (NARD Institute) was used. The acrylamide-pendant Phos-tag ligand (#AAL-107) and MnCl_2_ were added to 6% w/v acrylamide-gels in concentrations from 0 to 40 µM. Before blotting gels were equilibrated first in Blotting buffer (50 mM Tris, 45 mM Glycine, 0.04% SDS, 20% Methanol) containing 5 mM EDTA for 10 min and afterwards in Blotting buffer without EDTA for 10 min.

### Immunoprecipitation

For immunoprecipitation experiments, HeLa cells were plated in 75 cm^2^ flasks at a density of 8×10^5^ one day prior transfection. Cells were transfected with the respective plasmids using JetPEI transfection reagent (Polyplus Transfections) according to the manufacturer's instructions. 48 hours after transfection cells were lysed by sonication in IP-buffer [containing 50 mM Tris pH 7.5, 2.5 mM MgCl_2_, 100 mM NaCl, 1 mM DTT, PhosStop (phosphatase inhibitor cocktail; Roche), complete (protease inhibitor cocktail; Roche)]. Immunoprecipitation was carried out using Protein A-Agarose (Roche) following the manufacturer's instructions.

## Results

### Subcellular localization and transcriptional activity of GLI3 is regulated by the MID1-**α**4-PP2A complex

**Figure 1:**
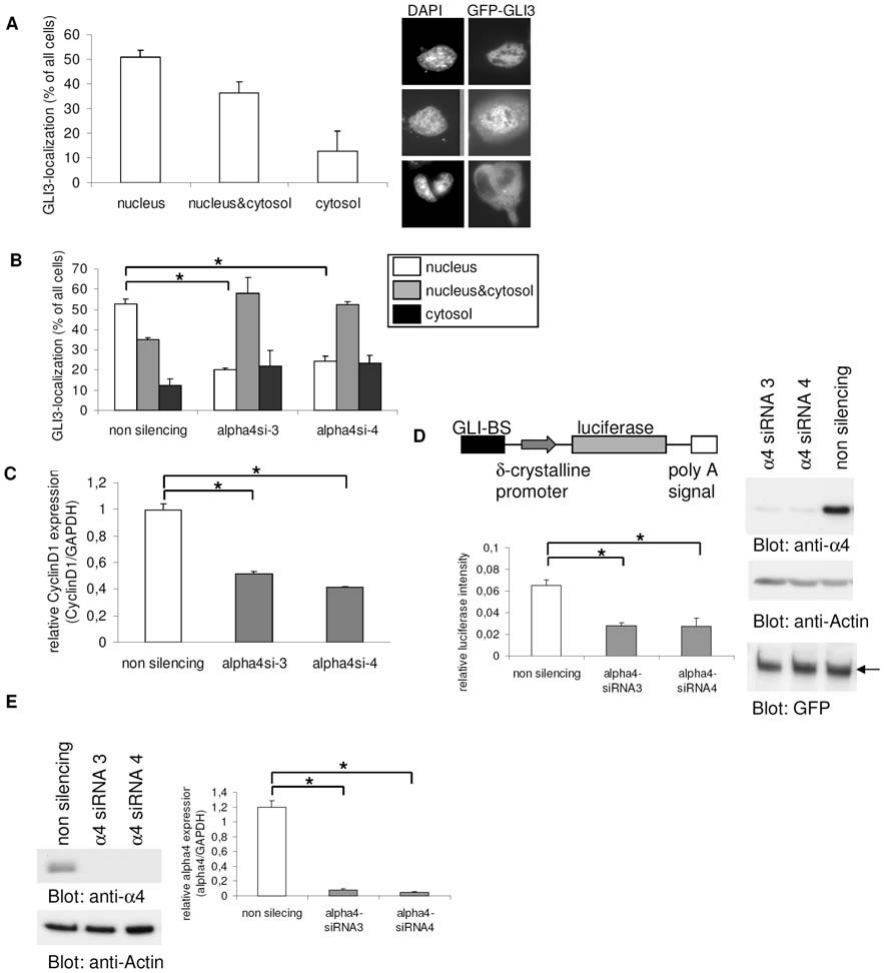
Subcellular localization and transcriptional activity of GLI3 after α4-knockdown. (a) Subcellular distribution of GFP-GLI3 in HeLa cells. Right: GFP signal distribution in individual cells occurred in three patterns: exclusively nuclear fluorescence (upper panel); even staining throughout the cytosol and nucleus (middle panel); or predominantly cytosolic fluorescence (lower panel). Left: Transfected cells were randomly chosen, counted and classified in one of the three groups. Data shown represent mean ± s.d. scored per group from 3 independent experiments of 100 cells each. (b) Subcellular distribution of GFP-GLI3 in HeLa cells after co-transfection with either non-silencing control siRNA's (left columns) or α4-specific siRNA's 3 and 4 (middle and right columns). The relative abundance of cells with exclusively nuclear GFP-GLI3 is shown in white columns, even staining throughout the cytosol and nucleus is shown in gray columns and black columns represent cells with predominantly cytosolic fluorescence. T-Test (two-tailed, homoscedastic): *p≤0.0001. (c) Relative *Cyclin D1* mRNA amounts in HeLa cells transfected with either non-silencing control siRNA's (white column) or α4-specific siRNA's 3 and 4 (gray columns) as measured by real-time PCR. Columns represent mean values of 4 samples measured in parallel +/− s.d. GAPDH was used for normalization. T-Test (two-tailed, homoscedastic): *p≤0.00000001 (d) GLI3-reporter assay. Left: Firefly-luciferase under the control of eight GLI-binding sites was co-transfected with α4- specific (gray columns) or control-siRNA's (white columns). As an internal transfection control renilla-luciferase was included and used for normalization. Columns show relative firefly-luciferase signals from 4 samples ± s.d. Right: Western blot of lysates from the GLI3-reporter assay. Similar expression levels of GFP-GLI3 in control and knock-down samples are shown by detection with anti-GFP antibodies (lower panel). Loading of equal protein amounts is shown by Actin-staining (middle panel). For knock-down control α4 was detected on the same membrane (upper panel). (e) Knock-down controls: Left: Knock-down efficiency of α4 (lanes 2 and 3) compared to a control treated with non-silencing siRNA's (lane 1) is shown on a Western blot using an anti-α4 antibody (upper panel). Loading of equal protein amounts is shown by Actin-staining (lower panel). Right: Effects of α4 siRNA's compared to a control treated with non-silencing siRNA's on the endogenous α4 mRNA level as measured by real-time PCR. Columns represent mean values of 4 samples measured in parallel +/− s.d. GAPDH was used for normalization. T-Test (two-tailed, homoscedastic): *p≤0.0000003.

**Figure 2:**
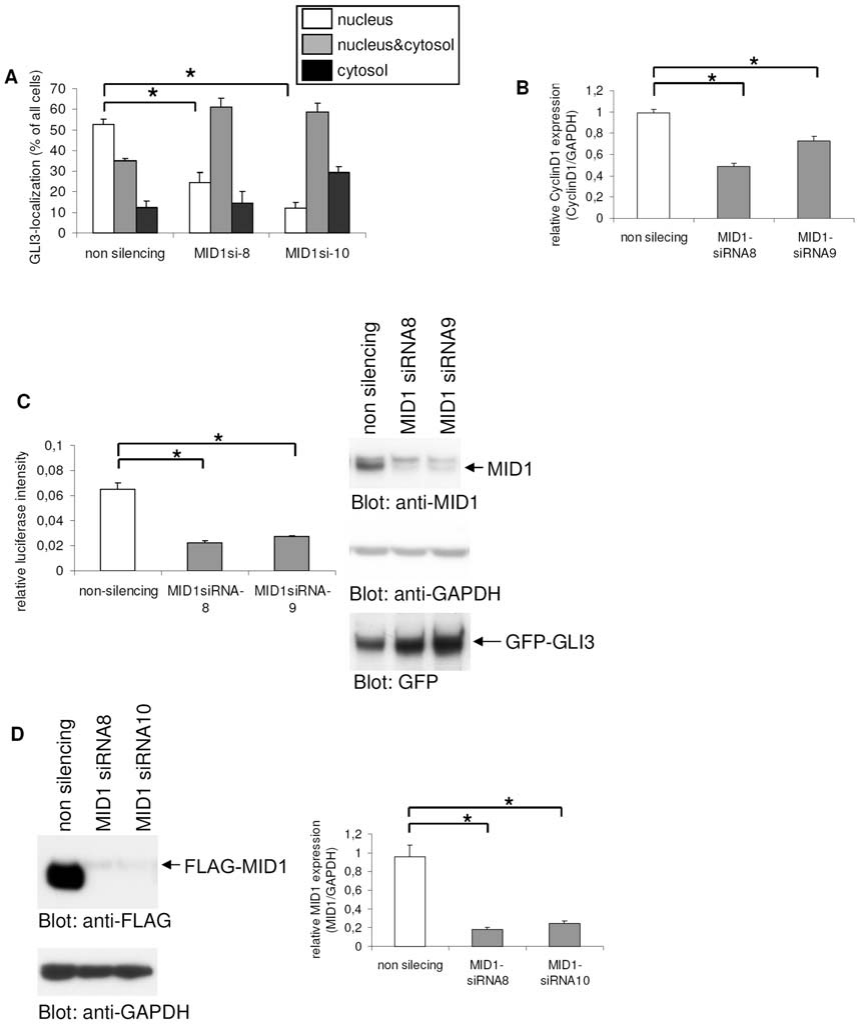
Subcellular localization and transcriptional activity of GLI3 after MID1-knockdown. (a) Subcellular distribution of GFP-GLI3 in HeLa cells after co-transfection with either non-silencing control siRNA's (left columns) or MID1-specific siRNA's 8 and 10 (middle and right columns). The relative abundance of cells with exclusively nuclear GFP-GLI3 is shown in white columns, even staining throughout the cytosol and nucleus is shown in gray columns and black columns represent cells with predominantly cytosolic fluorescence. T-Test (two-tailed, homoscedastic): *p≤0.001. (b) Relative mRNA amounts of *Cyclin D1* in HeLa cells transfected with either non-silencing control siRNA's (white column) or MID1-specific siRNA's 8 and 10 (gray columns) as measured by real-time PCR. Columns represent mean values of 4 samples measured in parallel +/− s.d. GAPDH was used for normalization. T-Test (two-tailed, homoscedastic): *p≤0.0002 (c) GLI3-reporter assay. Left: Firefly-luciferase under the control of eight GLI-binding sites was co-transfected with MID1- specific (gray columns) or control-siRNA's (white columns). As an internal transfection control renilla-luciferase was included and used for normalization. Columns show relative firefly-luciferase signals from 4 samples ± s.d. Right: Western blot of lysates from the GLI3-reporter assay. Similar expression levels of GFP-GLI3 in control and knock-down samples are shown by detection with anti-GFP antibodies (lower panel). Loading of equal protein amounts is shown by GAPDH-staining (middle panel). For knock-down control endogenous MID1 was detected on the same membrane (upper panel). (d) Knock-down controls: Left: Knock-down efficiency of MID1 specific (lanes 2 and 3) compared to non-silencing siRNA's (lane 1) on overexpressed Flag-tagged MID1 protein levels is shown on a Western blot using an anti-Flag antibody (upper panel). Loading of equal protein amounts is shown by GAPDH-staining (lower panel). Right: Effects of MID1 siRNA's compared to a control treated with non-silencing siRNA's on the endogenous MID1 mRNA level as measured by real-time PCR. Columns represent mean values of 4 samples measured in parallel +/− s.d. GAPDH was used for normalization. T-Test (two-tailed, homoscedastic): *p≤0.0002.

We have recently shown that the subcellular localization and transcriptional activity of GLI3 is regulated by PP2A activity and the MID1-α4 ubiquitin ligase complex in several cancer cell lines with autonomously activated SHH-signaling. Endogenous GLI3 is expressed in all these cell lines including HeLa (cervix carcinoma) and U373MG (glioblastoma). In confirmation of these results, we have now co-transfected GFP-GLI3 in HeLa cells either with MID1- or α4-specific siRNA oligonucleotides and analyzed its subcellular localization as described [18]. The transcriptional activity of GLI3 was monitored by real-time PCR of the GLI3 target *cyclin D1* and in a luciferase reporter assay. As shown in Fig. 1a, over-expressed C-terminally GFP-tagged GLI3 localized predominantly to the nucleus in 51% of transfected cells, exclusively in the cytosol in 13% of cells, and showed a diffuse pattern in 36% of transfected cells. To verify the precise localization of GFP-GLI3 and the proper co-detection of nuclei (DAPI) and nuclear protein we applied confocal microscopy, showing comparable localization patterns as detected by epifluorescence (Figure S1). This is in line with our previous observations [18]. Disruption of the MID1-α4-PP2A complex by RNAi-mediated knock-down of endogenous α4 caused a significant reduction in nuclear GLI3 localization (Fig. 1b). Concomitantly, cells with diffuse and strictly cytosolic GLI3 were increased. The cytosolic retention of GLI3 correlated with a reduced transcription of its target gene *cyclin D1* (Fig. 1c) and a decreased transcriptional activity of GLI3 in a GLI3-reporter assay (Fig. 1d). To check the specificity of the reporter assay for GLI3 transcriptional activity, we used GFP tagged GLI3-deletion constructs, which either lack the DNA-binding zinc-finger domains (constructs containing amino acids 1-396, 824-1100), or a construct containing only two out of the five zinc-finger domains (amino acids 568-1549). While only weak induction of the GLI3 reporter by the deletion constructs was observed, full-length GFP-GLI3 caused a significant increase in reporter activity (Figure S2). To rule out the possibility that the observed reduction of the GLI3-reporter after α4-knockdown was caused by destabilization of the GFP-GLI3 protein, GFP-GLI3 was detected on a Western blot, showing no differences in protein amounts after knockdown (Fig. 1d). The efficiency of the knock-down procedure was proven by Western blot detecting the α4 protein and Real-time-PCR amplifying the α4 mRNA (Fig. 1e). Comparable results were obtained using different, non-overlapping siRNA molecules and with N-terminally tagged GLI3 constructs, indicating that the observed localization effects are independent of target sequence and protein cleavage (Table S2). Similarly, nuclear GFP-GLI3 was also reduced after knock-down of the MID1 protein (Fig. 2a). Again, cytosolic retention of GLI3 resulted in a decreased transcriptional activity on its target gene *cyclin D1* (Fig. 2b) and in a reduced GLI3-reporter activity, without reduction of overall GFP-GLI3 protein levels (Fig. 2c). Knockdown efficiency was analyzed by Western blot and Real-time-PCR (Fig. 2e).

### Subcellular localization of mutant GLI3 proteins

**Figure 3:**
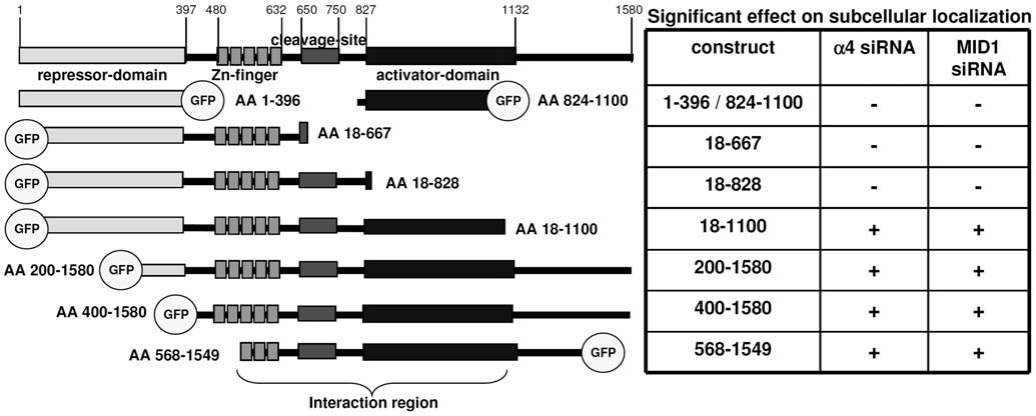
Mapping of the GLI3-protein region critical for it's MID1-PP2A-dependent regulation. Left: Schematic representation of the functional domains of GLI3 and the deletion constructs used in this study. Right: Effects of MID1- or α4-knockdown on the nuclear localization of the respective GLI3-deletion-construct. A significant decrease of nuclear GLI3 is indicated by +.

In order to narrow down the protein region critical for MID1-PP2A-dependent regulation of GLI3, we co-transfected several GLI3 deletion mutants together with α4- or MID1-specific siRNA's. Using this approach, we have mapped the functional interaction between the MID1-α4-PP2A complex and GLI3 to a region between amino acid 568 and 1100 (Fig. 3).

**Figure 4:**
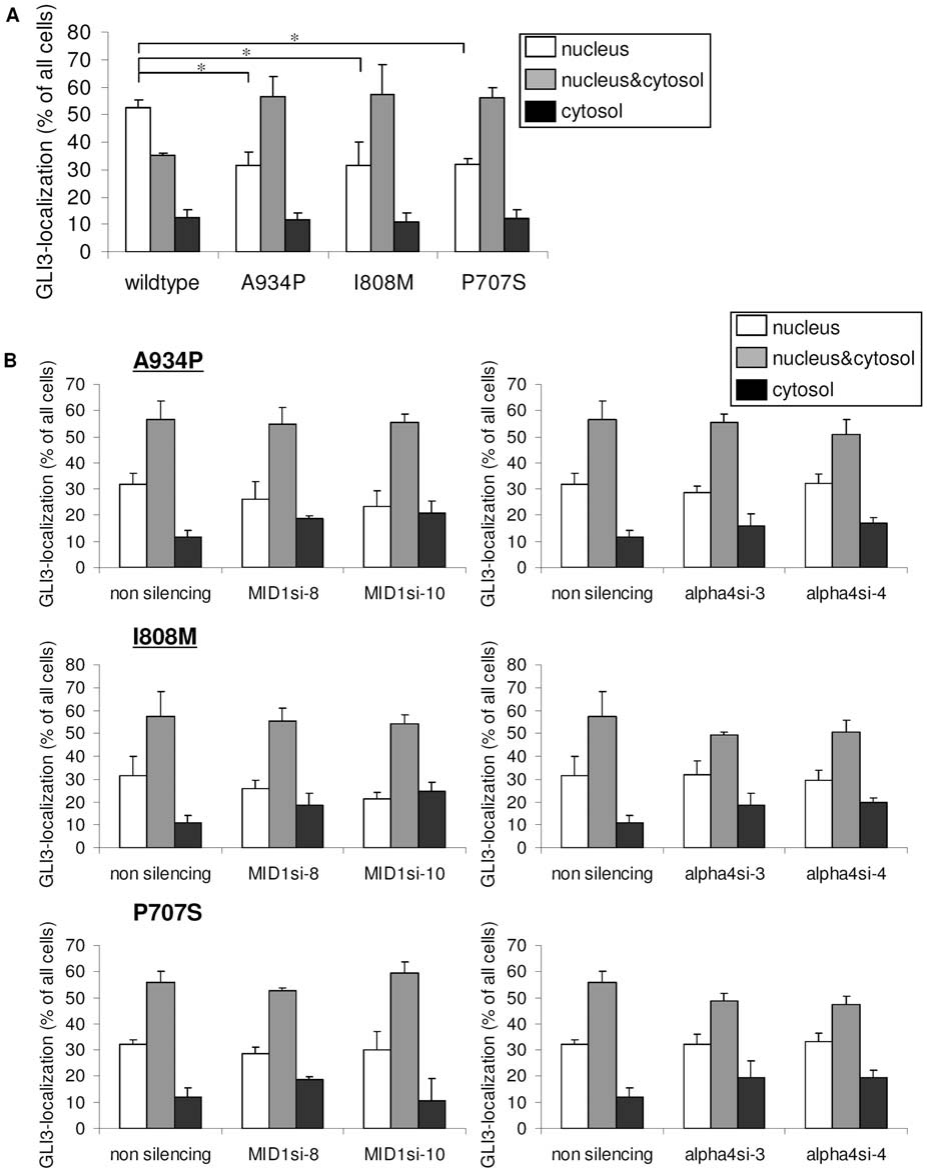
Subcellular localization of mutant GFP-GLI3. (a) Subcellular distribution of wt and mutant GFP-GLI3 in HeLa cells. Visualization and scoring were performed exactly as described in the legend to Fig. 1. The relative abundance of cells with exclusively nuclear GFP-GLI3 is shown in white columns, even staining throughout the cytosol and nucleus is shown in gray columns and black columns represent cells with predominantly cytosolic fluorescence. Data shown represent mean ± s.d. scored per group from 3 independent experiments of 100 cells each. T-Test (two-tailed, homoscedastic): *p≤0.015. (b) Subcellular distribution of mutant GFP-GLI3 (upper panel mutant A934P, middle panel mutant I808M, lower panel mutant P707S) in HeLa cells after cotransfection with α4- (right panel) or MID1-specific (left panel) siRNA's. Visualization and scoring were performed exactly as described in the legend to Fig. 1. The relative abundance of cells with exclusively nuclear GFP-GLI3 is shown in white columns, even staining throughout the cytosol and nucleus is shown in gray columns and black columns represent cells with predominantly cytosolic fluorescence. Data shown represent mean ± s.d. scored per group from 3 independent experiments of 100 cells each. T-Test (two-tailed, homoscedastic): (p>0.1).

Interestingly, some GCPS point mutations and a mutation in a patient with acrocallosal syndrome are located in the identified MID1-α4-PP2A complex-dependent regulation domain of GLI3 [A943P (as found in one patient with acrocallosal syndrome [15]), I808M [11] and P707S [17] (as found in GCPS patients)]. These point mutations could lead to a loss of the MID1-α4-PP2A complex-regulated nuclear accumulation of GLI3. In order to confirm this hypothesis, we introduced the three mutations into the GFP-GLI3 protein and analyzed the mutant proteins for their subcellular localizations. Compared to wt GFP-GLI3, a significant reduction of cells with predominantly nuclear GFP-GLI3 localization (approximately 53% of wt GLI3 compared to 32% of the mutants P707S, I808M, and A934P) was observed (Fig. 4a) in HeLa cells. The same effect was seen in U373MG cells (Figure S3). These results strongly suggest that the point mutations indeed induce a misregulation of the subcellular localization of GLI3. Furthermore, none of the mutant proteins reacted like wt GLI3 upon α4 or MID1 knock-down in HeLa cells (Fig. 4b). Again, similar results were obtained in U373MG cells (Figure S4).

### Point mutations lead to a reduced transcriptional activity of GLI3

**Figure 5:**
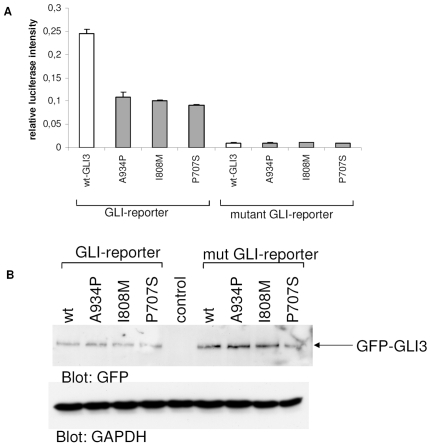
GLI3-reporter assay. (a) Firefly-luciferase under the control of eight GLI-binding sites (normal or mutant for negative controls) was co-transfected either with wt (white columns) or mutant (gray columns) GFP-GLI3. As an internal transfection control renilla-luciferase was included and used for normalization. Columns show relative firefly-luciferase signals from 4 samples ± s.d. (b) Western blot of lysates from (a). Similar expression levels of wt and mutant GFP-GLI3 are shown by detection with anti-GFP antibodies (upper panel). Loading of equal protein amounts is shown by GAPDH-staining (lower panel).

Misregulation of the subcellular localization of mutant GLI3 would be consistent with a reduction of its transcriptional activity. In order to analyze effects of the point mutations on GLI3 transcriptional activity, we co-transfected GLI3 reporter plasmid carrying either wild-type or mutated GLI binding sites with either wt or mutant GFP-GLI3. Interestingly, the mutant GLI3 proteins had significantly decreased capabilities to induce the luciferase signal, suggesting that indeed the misregulated localization of mutant GLI3 leads to a decrease in its transcriptional activity (Fig. 5a). Equal transfection and synthesis efficiencies of the wt and mutant GLI3-constructs were controlled on a Western blot analyzing ratios of expressed GLI3 to GAPDH (Fig. 5b).

### GLI3 partially colocalizes with the microtubule-associated MID1-protein

**Figure 6:**
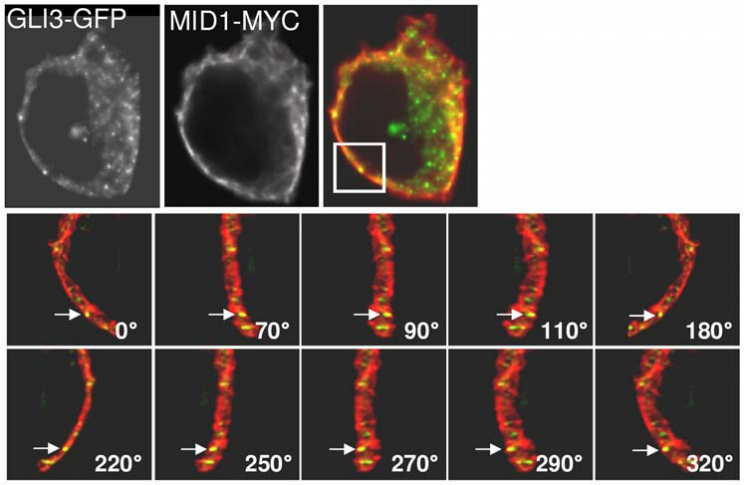
Colocalization of GFP-GLI3 and myc-MID1. Upper panel: Representative section of a HeLa cell cotransfected with GFP-GLI3 and myc-MID1. A single focal plane of a deconvoluted 3D-image stack is shown. Yellow colour represents colocalization. Lower panel: Higher magnification of the labelled sector from the upper panel and view from different angles to show that colocalization of GFP-GLI3 and myc-MID1 does not result from random overlapping. Arrows point to the same signal in every picture. Angles are defined as indicated in the pictures.

Since we could show that subcellular GLI3-localization was dependent on the microtubule-associated MID1-α4-PP2A complex, we tested whether a co-localization of GLI3 and the microtubules-associated MID1-protein could be detected by 3D-immunofluorescence. GFP-tagged GLI3 and myc-tagged MID1 were co-expressed in HeLa cells and visualized by Immunofluorescence. Results show a locally restricted co-localization of GFP-GLI3 and MID1-myc (Fig. 6) indicating a transient proximity of both.

### Phosphorylation of GLI3

**Figure 7:**
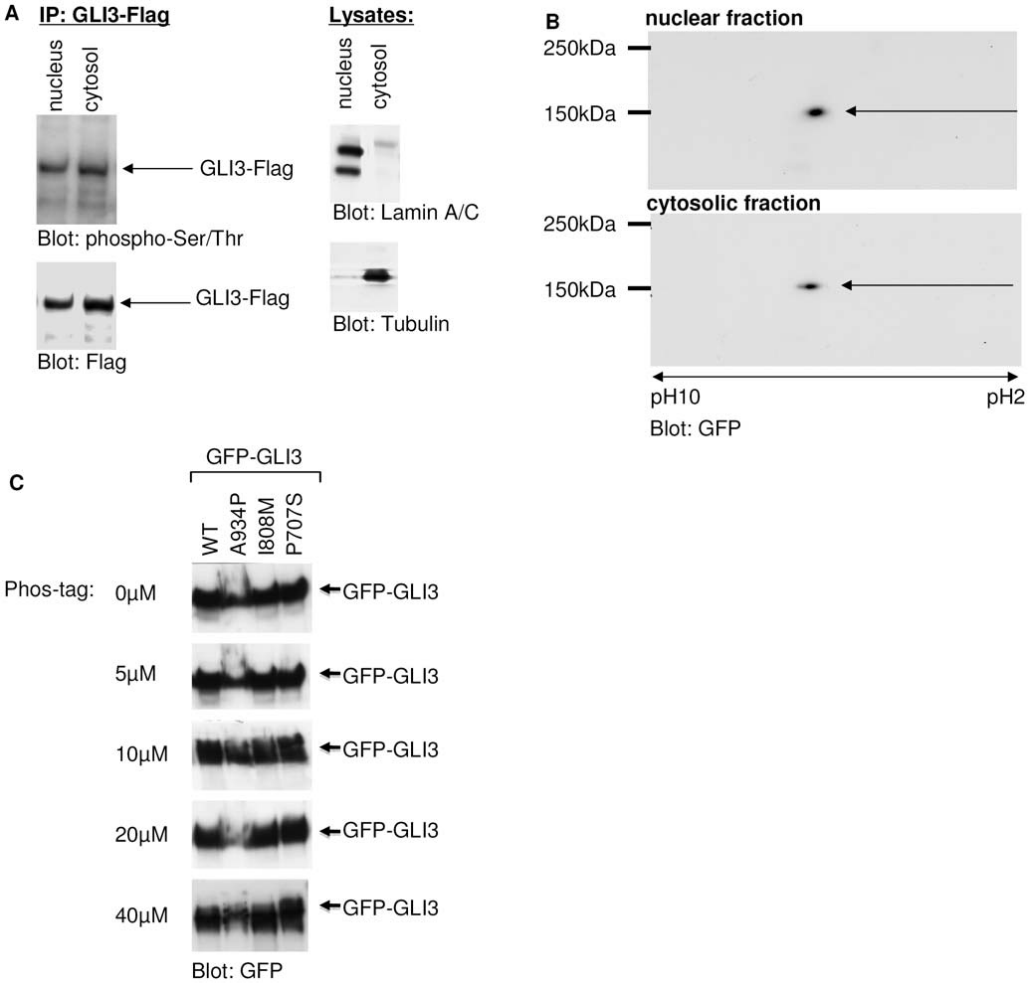
Phosphorylation of GLI3. (a) Left: GLI3-Flag was expressed in HeLa cells and isolated from nuclear or cytosolic fractions by immunoprecipitation. Immunoprecipitates were analyzed by Western blot. Phosphorylation of GLI3-Flag was visualized by staining with a pool of phospho-serine and phospho-threonine antibodies (upper panel). Afterwards the same membranes were stripped and incubated with Flag-antibodies to verify that the phospho-specific bands co-stain with GLI3-flag (lower panel). Right: To control the efficiency of the fractionation procedure, aliquots of the nuclear and cytosolic fractions were analyzed on Western blots with anti-LaminA/C- and anti-Tubulin-antibodies. (b) Nuclear and cytosolic fractions of GFP-GLI3-AA568-1549 over-expressing HeLa cells were separated on 2D-Gels and blotted with an anti-GFP-antibody. (c) Different concentrations of the acrylamide-pendant Phos-tag ligand (0, 5, 10, 20, 40 µM) have been added to 6% SDS-Gels that were used to analyse GFP-GLI3, wild-type (wt) and mutant (A934P, I808M, P707S). An anti-GFP antibody has been used for detection.

Changes in PP2A activity result in misregulation of both - the subcellular localization and transcriptional activity of GLI3. This regulation could be mediated either by phosphorylation of the GLI3 molecule itself (which would predict a different phosphorylation pattern of nuclear and cytosolic GLI3) or by phosphorylation of one of its interaction partners. To distinguish between the two possibilities we analyzed the phosphorylation status of GLI3. In a first set of experiments we tested if GLI3 is differentially phosphorylated in dependency of its localization to the cytosol or the nucleus by expressing Flag-GLI3 in HeLa cells. Nuclear and cytosolic fractions were prepared, immunoprecipitated and analyzed with a cocktail of anti-phospho-serine and -threonine antibodies. As shown in Fig. 7a, serine/threonine phosphorylation was detectable in both, the nuclear and cytosolic fraction. Purity of the fractions was controlled by lamin A/C and tubulin detection. To detect eventually differing phosphorylation patterns between nuclear and cytosolic GLI3, 2D PAGE and Phos-Tag-SDS-PAGE were performed: The isoelectric focusing pattern obtained in a 2D PAGE analysis, which is highly sensitive to changes in phosphorylation, showed an identical phosphorylation status of a GFP-GLI3-construct (amino acids 568-1549) containing the PP2A-dependent regulatory domain in both cellular compartments (Fig. 7b). These results were confirmed by the analysis of total lysates of full-length GFP-GLI3 expressing cells using the Phos-Tag-technology, by which differentially phosphorylated protein-isoforms can be separated by their molecular weight. On gels containing increasing concentrations of the acrylamide-pendant Phos-tag ligand only one GLI3 band was detected suggesting that GLI3 is a mono-phosphorylated protein in the cell system used, where PKA is not stimulated. Furthermore the separation of the wt and mutant GFP-GLI3 constructs on Phos-tag-gels showed no differences between wt and mutant GLI3-proteins (Fig. 7c). Taken together these data strongly argue against the PP2A-dependent regulation of GLI3 localization by its own phosphorylation pattern, thereby suggesting the involvement of phospho-modified co-factors.

## Discussion

In this study, we demonstrate that the SHH target GLI3 is regulated by a functional interaction between a MID1-α4-PP2A complex-dependent regulation domain of GLI3 and the MID1-α4-PP2A complex. Increased PP2A activity mediated by MID1 or α4 knock-down resulted in the cytosolic retention of full-length GLI3 and led to a reduced transcriptional activity. Interestingly, GCPS- and acrocallosal syndrome-associated point mutations within the MID1-α4-PP2A complex-dependent interaction domain led to a misregulation of the subcellular localization, a loss of the MID1-α4-PP2A complex-dependent nuclear localization of GLI3, and a reduction of the transcriptional activity of mutant GLI3. As observed previously [18], the described mechanism affects primarily the GLI3 transcriptional activator in our model system.

### The MID1-**α**4-PP2A complex regulates the nuclear localization and transcriptional activity of the GLI3 activator

In this study, we have analyzed a functional interaction between the MID1-α4-PP2A complex and the transcription factor GLI3. In line with our previous results [18], changes in the subcellular localization of GLI3 correlated changes in its transcriptional activity. Transcriptional activity of GLI3 was measured by real-time PCR analysis of the GLI3 target gene *cyclin D1* and in a luciferase reporter assay driven by a GLI3-dependent promoter. Like its *Drosophila* homologue, Ci, GLI3 is known to be cleaved into its repressor form in absence of SHH, whereas SHH signaling represses cleavage, thereby promoting formation of full-length GLI3 [6], [22], [23]. While the importance of the GLI3 repressor in antagonizing SHH signaling has been well established, the role of GLI3 as a transcriptional activator has been more controversially discussed [23]–[28]. However, recent studies in mice clearly establish an important role for the GLI3 activator during development [8], [29]–[33]. In order to be able to distinguish between effects of the GLI3 activator and those of the GLI3 repressor peptides, we have chosen a tissue culture model in which GLI3 cleavage is reduced to a minimum and in which we had shown autonomous SHH activation [18]. In this model system, changes in the subcellular localization of GLI3 correlated with changes of its transcriptional activity: a decrease of nuclear GLI3 protein led to a reduction of transcriptional activity. In addition, transfection of a luciferase reporter under the control of a GLI3-dependent promoter together with wild-type GLI3 resulted in an increase in reporter activity, while mutant proteins led to a much smaller induction of the reporter, and knock-down of GLI3 in the model system led to a reduction of target expression [18]. Furthermore neither of the two deletion constructs that are quite similar to a putative repressor form (Figure 3, constructs 18-667 and 18-828) react on MID1 or α4 knock-down, which suggests that an intact PP2A-dependent regulatory domain is necessary for MID1-α4-PP2A complex regulation of the protein. In summary our data suggest that the observed PP2A-MID1-dependent regulatory mechanism primarily affects the GLI3 activator form.

### The MID1-**α**4-PP2A complex functionally interacts with amino acids 568-1100 of GLI3

Several functional domains have been described for the GLI3 protein. The N-terminus (amino acids 1-397) comprises the repressor domain [22], followed by five zinc finger domains (amino acids 480-632) and the cleavage site (amino acids 700-740) [6], [22]. An activation domain, which is responsible for the binding of GLI3 to its co-activator CBP (CREB binding protein), spans amino acids 827-1132 and includes multiple phosphorylation sites [22]. Phosphorylation of amino acids 849, 865, 877, 907, 980 and 1106 by PKA and amino acids 861, 873 and 903 byGSK3β leads to ubiquitination of GLI3 by SCF^βTrCP^, followed by N-terminal cleavage via the proteasome [9]. The PDD (processing determinant domain), which is necessary for efficient cleavage, spans the region from amino acid 648-915 [34]. The C-terminal end of the GLI3 protein is characterized by the MBD (Mediator binding domain), which links the full-length GLI3 to the Mediator complex [35]. We have now mapped a PP2A-dependent regulation domain to a region between amino acids 568-1100. Regulation of GLI3 by the MID1-α4-PP2A complex could be mediated either by modifying phosphorylation of GLI3 in its PP2A-dependent regulation domain or by the phospho-specific modification of a GLI3 cofactor binding to that region. All our data however suggest PP2A-dependent modification of a GLI3 cofactor: (i) No differences in the phosphorylation pattern between nuclear and cytosolic GLI3 could be detected either on one-dimensional SDS-Gels with anti-serine and anti-threonine antibodies as well as on 2D-gels, (ii) analysis of wild-type and mutants GLI3 on Phos-tag gels suggest only one (probably phosphorylated) GLI3 isoform in cells expressing either wild-type or mutants GLI3. However, further studies are required to identify this GLI3 cofactor.

### C-terminal point mutations in GLI3 cause the same phenotype as loss-of-function alleles

Mutations in the transcription factor GLI3 have been associated with several phenotypes, including GCPS, PHS and - in one patient - acrocallosal syndrome. While, loss-of-function mutations that lead to a reduction of GLI3 protein and haploinsufficiency in the patients are responsible for GCPS [5], [12], [17], truncating mutations resulting in dominant negative effects and gain-of-function of the repressor peptide cause PHS [6], [16]. However, some GCPS point mutations were discovered that do not lead to an obvious loss of the protein, but rather produce a mutated protein [11], [13]. Until now, no clear mechanism by which a point mutation in the C-terminal part of the protein could lead to the GCPS loss-of function phenotype has been described.

Here, we show that the subcellular localization of GLI3 and its transcriptional activity are regulated by the MID1-α4-PP2A complex. We have mapped the domain that is responsible for this regulatory interference to a novel PP2A-dependent regulation domain on the GLI3 protein between amino acid 568 and 1100. Some of the GCPS-associated point mutations in GLI3 localize to this region. Analysis of some of these point mutants for their intracellular localization pattern revealed that the mutant GLI3 proteins show abnormal subcellular localization and reduced transcriptional activity. Furthermore, the PP2A-dependent regulation of the subcellular localization was modified in the mutant GLI3 proteins. Since the nuclear localization of full-length GLI3 protein is directly linked to its capacity as transcriptional activator, the abnormal localization patterns of the mutant proteins point towards a loss of nuclear GLI3 with subsequent decreased activator function of the mutant GLI3 in patients.

### Related ventral midline syndromes share disturbed GLI3 localization

**Figure 8:**
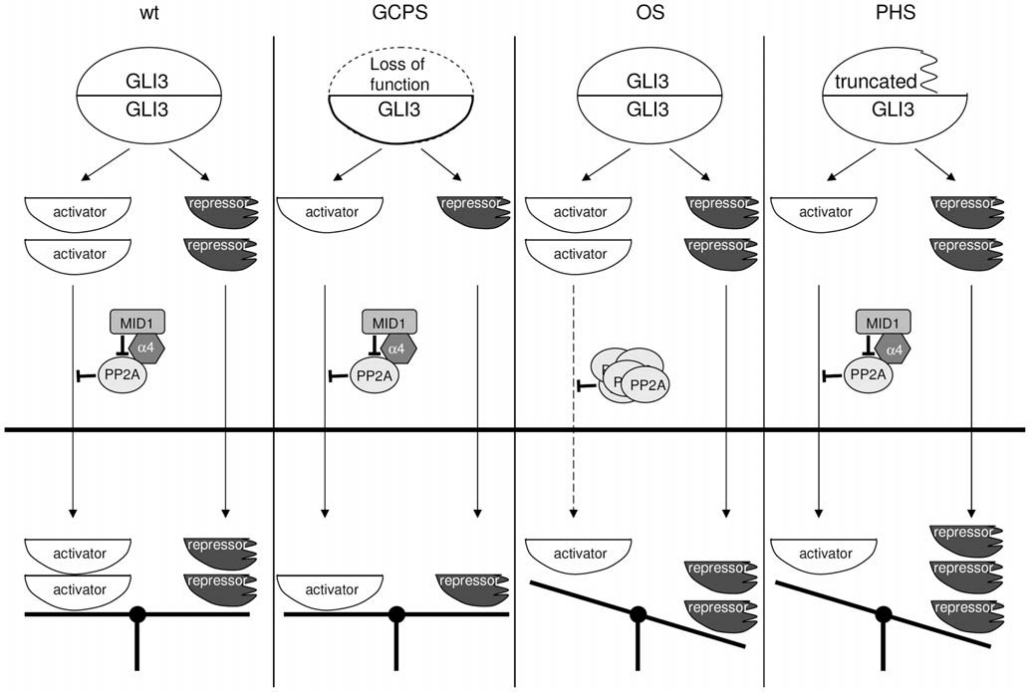
Hypothetical model. From left to right: Normal situation: The activity of the MID1-α4-PP2A complex controls the maturation of GLI3 into a transcriptional activator. Transcription of SHH targets depends on an equilibrium between GLI3 activator and GLI3 repressor. Situation in GCPS: One GLI3 allele is mutated to a loss of function allele. This leads to a decrease of the total amount of GLI3 activator and repressor molecules. Situation in OS: Loss-of-function of MID1 leads to hyperactivity of PP2A and to a reduction of nuclear GLI3 activator molecules. Apart from that, since the GLI3-repressor is not influenced by this misregulation, disequilibrium between repressor and activator in favour of the repressor occurs in the nucleus. Situation in PHS: Mutations in one GLI3 allele lead to the production of truncated protein with exclusively repressive properties from that allele. This leads to a relative excess of GLI3 repressor molecules relative to activator in the nucleus.

As suggested previously (reviewed by [36]) phenotypic overlap of syndromes in many cases point at shared molecular pathways of the genes responsible for the syndromes, which makes phenotype comparisons of monogenic syndromes a powerful tool to unravel biochemical relationships. Our results suggest a molecular mechanism underlying both the clinical similarities, as well as the disparities, between three developmental disorders, OS, caused by loss-of-function mutations in MID1 [20], GCPS, caused by loss-of-function in one GLI3 allele [11], [12], and PHS, caused by expression of truncated GLI3 due to premature stop codon formation [5], [13], [14]. Clinically, patients with all three syndromes show disturbed midline development which, in the so-called gliopathies (GCPS, PHS) is associated with limb malformations. Patients with OS may present with hypertelorism, broad nasal bridge, cleft lip and palate, laryngotracheal clefts, anal defects, and hypospadias. GCPS patients may show symptoms like hypertelorism, broad nasal root, and limb abnormalities such as pre- and postaxial polydactyly and syndactyly. PHS patients may present with cleft lip, palate or uvula, laryngeal clefts and tracheal defects, anal defects, and limb malformations like syn-, poly- or oligodactyly. The net cellular response to SHH signaling appears to be controlled by the relative abundance of full-length, transcriptionally active GLI3 and a transcriptionally repressive GLI3 cleavage fragment [5], [8], [10], [29], [33]. Our data show that full-length GLI3, but not the N-terminal repressor peptide, is regulated by MID1-PP2A. Based on the molecular aberrations underlying each phenotype, the present data allow the formulation of a unifying hypothesis to account for the overlap, but also some disparities between the three syndromes (Fig. 8). In GCPS, one full-length GLI3 allele is lost, causing a reduction of both activator and repressor, resulting in apparent haploinsufficiency of GLI3 activator and repressor [11]. In PHS, truncating mutations cause excess formation of repressor peptide, which acts in a dominant-negative manner [5], [13]. In OS, mutations in MID1 cause reduced nuclear activator GLI3 products, resulting in overlap with the haploinsufficiency in GCPS. Since only the full-length activator form is removed from the nucleus, the net effect in OS is also an excess nuclear abundance of GLI3 repressor, yielding phenotypic overlap with PHS. Thus, while mutations in the target protein GLI3 result in diverse phenotypes depending on the location of the respective mutation, dysfunction of MID1 leads to a ‘compound’ syndrome. Interestingly, in an analysis using fully automated text mining, Brunner et al. [36] have identified OS as the second nearest phenotypic neighbor of PHS. Our data now fill the gap between the phenotypic overlap of the two syndromes and possible underlying molecular mechanisms by suggesting a biochemical crosstalk between the involved gene products. In contrast to the shared midline phenotypes, limb development is affected by loss-of-function of GLI3 but rarely by MID1 mutations. This difference might be either due to the fact that polydactyly phenotypes, as seen in GCPS, are primarily caused by a loss of GLI3-repressor, the regulation of which might be unaffected by the MID1-α4-PP2A complex or it might reflect a fine-tuned, tissue-specific regulation of the MID1 function and/or a compensation of MID1 function by MID2 [19], [37]–[40] in some tissues. Thus, it might be that, due to as yet undefined mechanisms, tissues that participate to the development of ventral midline structures and their abnormalities are particularly sensitive towards MID1 dysfunction. An important structure that might be involved in the development of OS, GCPS and PHS phenotypes are primary cilia. Their close association with microtubules and growing evidence that intraflagellar transport and cilia development play an important role in GLI3-processing and -signalling [41], [42], suggest that the MID1-α4-PP2A protein complex, which is involved in microtubule-associated transport [43], could play a role in the establishment or function of this structure. However, the accumulation of PP2Ac in OS cells most likely does not lead to a complete retention of GLI3 in the cytosol and, while insufficient for the establishment of ventral midline structures, the residual nuclear activity of GLI3 supports normal limb development.

In summary, our results give insight into a thus-far unknown regulatory mechanism involving the MID1-α4-PP2A complex and GLI3 in a human cell line model. However, further in vivo studies are required to analyze the temporal and local regulation of these interactions during development.

## Supporting Information

Figure S1 Subcellular distribution of GFP-GLI3 in HeLa cells as determined by confocal microscopy. GFP signal distribution in individual cells occurred in three patterns: exclusively nuclear fluorescence (e.g. as in cells marked by arrows, upper panel); even staining throughout the cytosol and nucleus (e.g. as in cells marked by stars, lower panel); or predominantly cytosolic fluorescence (e.g. as in cells marked by arrowheads, upper panel). DAPI staining was used to label nuclei.
(5.71 MB TIF)

Figure S2 GLI3-reporter assay. Firefly-luciferase under the control of eight GLI-binding sites (normal or mutant for negative controls) was co-transfected either with full-length GFP-GLI3 (amino acids 18-1549) or GFP-GLI3 deletion constructs (amino acids 1-396, 824-1100, or 568-1549). As an internal transfection control renilla-luciferase was included and used for normalization. The relative GLI3-reporter induction by over-expression of the respective GFP-GLI3 constructs normalized to the respective signal of the mutant GLI3-reporter is shown. Columns represent signals from 3 samples ± st.dev.
(0.98 MB TIF)

Figure S3 Subcellular distribution of wildtype and mutant GFP-GLI3 in U373MG cells. Visualization and scoring were performed exactly as described in the legend to Fig. 1. The relative abundance of cells with exclusively nuclear GFP-GLI3 is shown in white columns, even staining throughout the cytosol and nucleus is shown in gray columns and black columns represent cells with predominantly cytosolic fluorescence. Data shown represent mean ± s.d. scored per group from 3 independent experiments of 100 cells each. T-Test (two-tailed, homoscedastic): *p<0.0005.
(1.39 MB TIF)

Figure S4 Subcellular distribution of mutant GFP-GLI3 (upper panel mutant A934P, middle panel mutant I808M, lower panel mutant P707S) in U373MG cells after cotransfection with alpha4- or MID1-specific siRNA's. Visualization and scoring were performed exactly as described in the legend to Fig. 1. The relative abundance of cells with exclusively nuclear GFP-GLI3 is shown in white columns, even staining throughout the cytosol and nucleus is shown in gray columns and black columns represent cells with predominantly cytosolic fluorescence. Data shown represent mean ± s.d. scored per group from 3 independent experiments of 100 cells each.
(2.17 MB TIF)

Table S1 Table S1a: Sequences of siRNA's used in this study. Table S1b: Sequences of primers used for real-time PCR experiments.
(0.05 MB DOC)

Table S2 Table S2 Summary of the subcellular localization of GFP-GLI3 (N-terminally tagged) and GLI3-GFP (C-terminally tagged) after knock-down of alpha4 using different siRNAs. Visualization and scoring were performed exactly as described in the legend to Fig. 1.
(0.04 MB DOC)
