# Supplementary figures and images for: Point Mutations in GLI3 Lead to Misregulation of its Subcellular Localization
Source: PLoS One. 2009 Oct 15;4(10):e7471. doi: 10.1371/journal.pone.0007471 (PMC2758996; doi:10.1371/journal.pone.0007471)

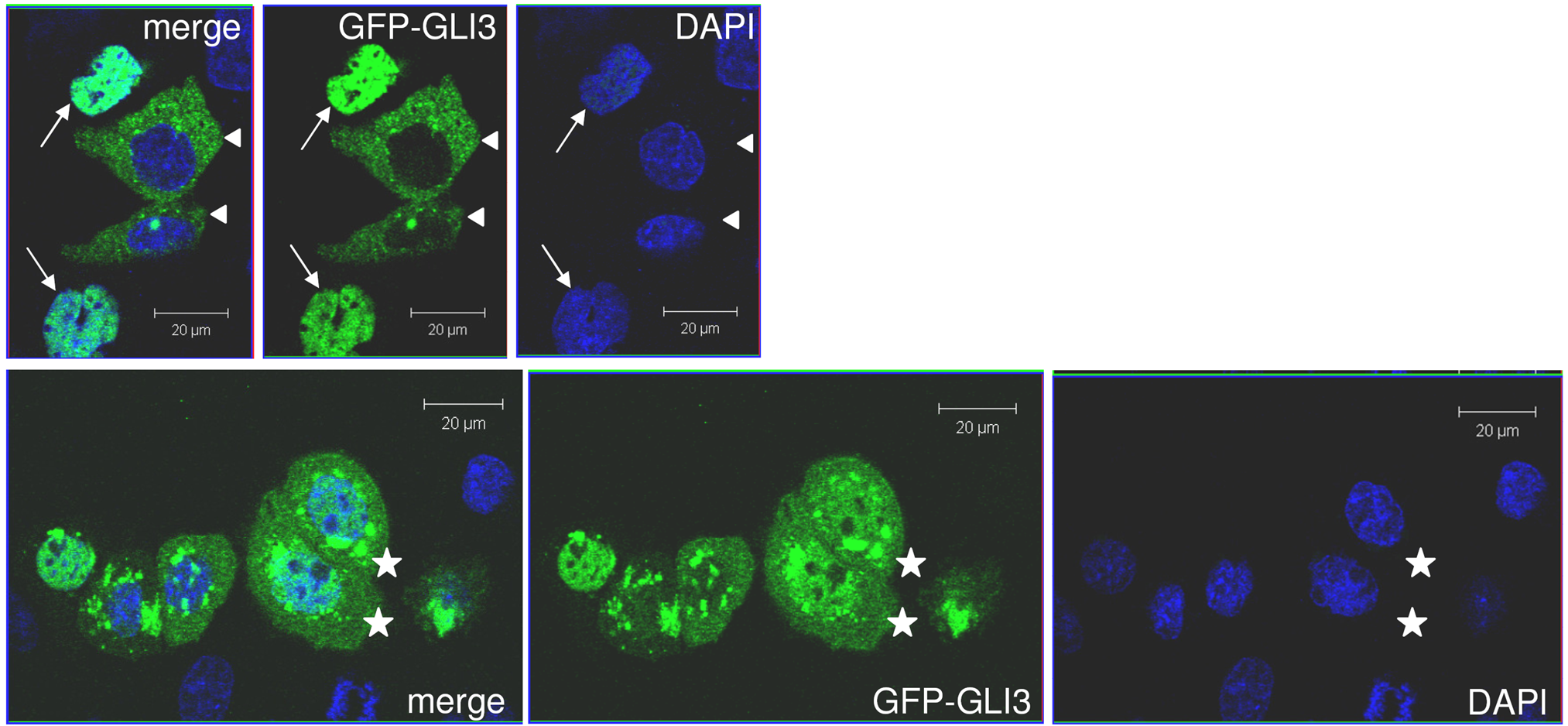

Supplement: Figure S1 — Subcellular distribution of GFP-GLI3 in HeLa cells as determined by confocal microscopy. GFP signal distribution in individual cells occurred in three patterns: exclusively nuclear fluorescence (e.g. as in cells marked by arrows, upper panel); even staining throughout the cytosol and nucleus (e.g. as in cells marked by stars, lower panel); or predominantly cytosolic fluorescence (e.g. as in cells marked by arrowheads, upper panel). DAPI staining was used to label nuclei. (5.71 MB TIF) [file pone.0007471.s001.tif]

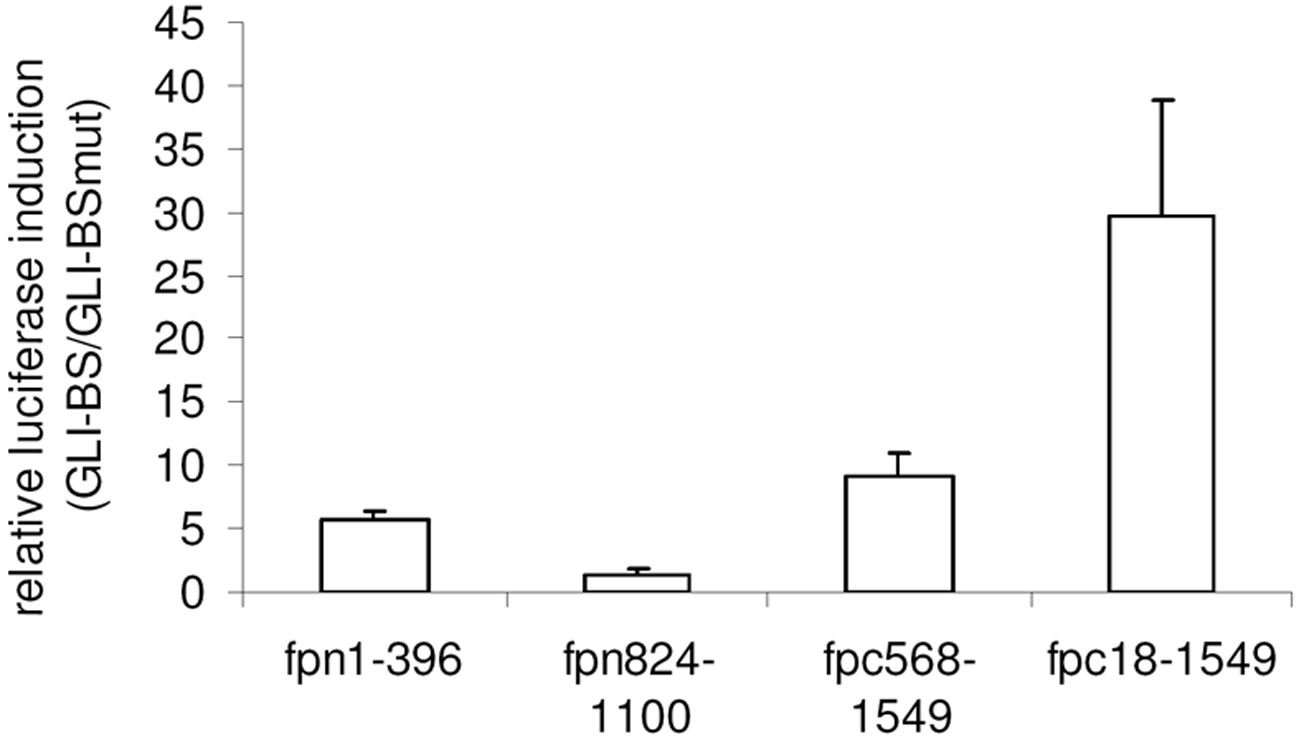

Supplement: Figure S2 — GLI3-reporter assay. Firefly-luciferase under the control of eight GLI-binding sites (normal or mutant for negative controls) was co-transfected either with full-length GFP-GLI3 (amino acids 18-1549) or GFP-GLI3 deletion constructs (amino acids 1-396, 824-1100, or 568-1549). As an internal transfection control renilla-luciferase was included and used for normalization. The relative GLI3-reporter induction by over-expression of the respective GFP-GLI3 constructs normalized to the respective signal of the mutant GLI3-reporter is shown. Columns represent signals from 3 samples ± st.dev. (0.98 MB TIF) [file pone.0007471.s002.tif]

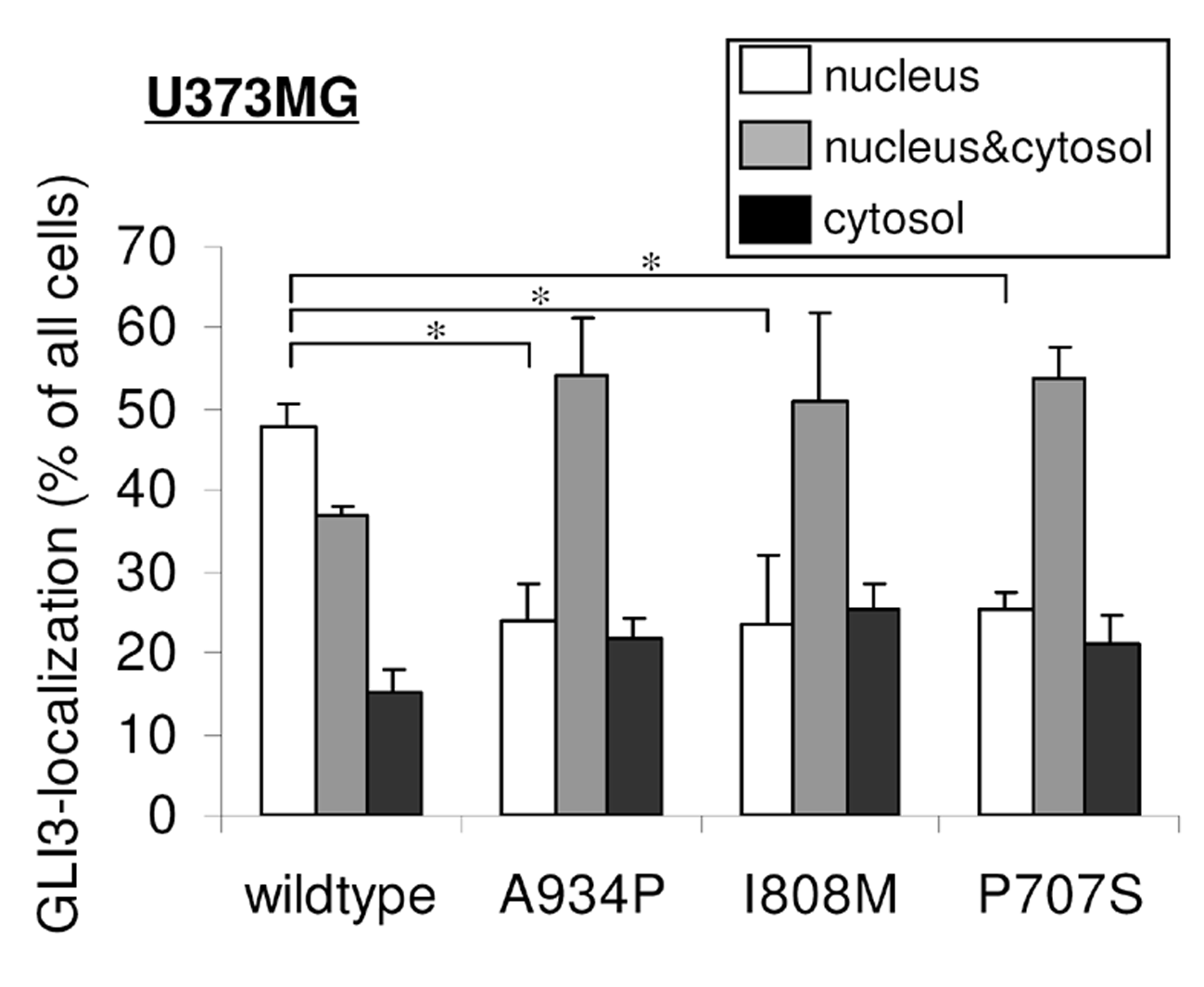

Supplement: Figure S3 — Subcellular distribution of wildtype and mutant GFP-GLI3 in U373MG cells. Visualization and scoring were performed exactly as described in the legend to Fig. 1. The relative abundance of cells with exclusively nuclear GFP-GLI3 is shown in white columns, even staining throughout the cytosol and nucleus is shown in gray columns and black columns represent cells with predominantly cytosolic fluorescence. Data shown represent mean ± s.d. scored per group from 3 independent experiments of 100 cells each. T-Test (two-tailed, homoscedastic): *p<0.0005. (1.39 MB TIF) [file pone.0007471.s003.tif]

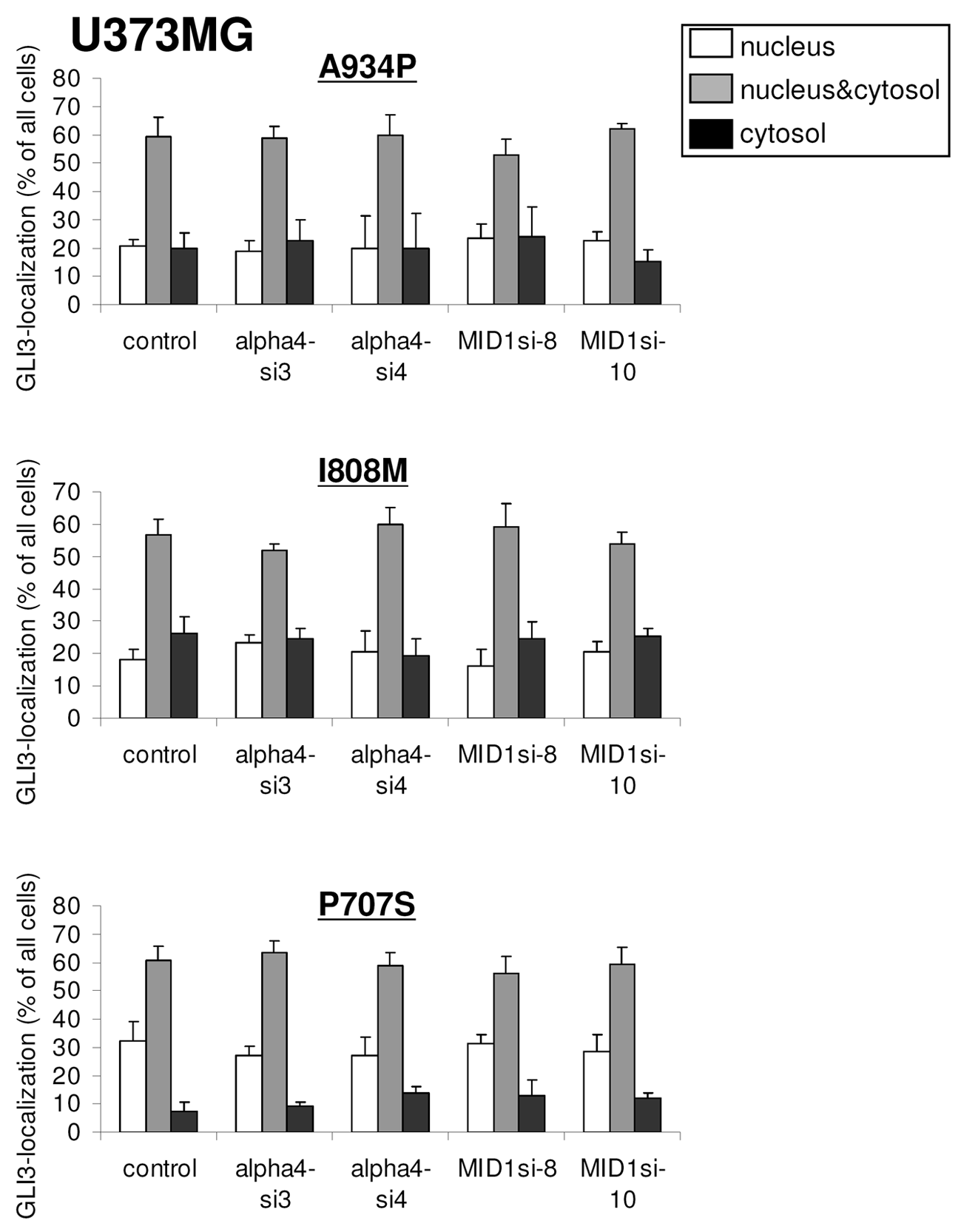

Supplement: Figure S4 — Subcellular distribution of mutant GFP-GLI3 (upper panel mutant A934P, middle panel mutant I808M, lower panel mutant P707S) in U373MG cells after cotransfection with alpha4- or MID1-specific siRNA's. Visualization and scoring were performed exactly as described in the legend to Fig. 1. The relative abundance of cells with exclusively nuclear GFP-GLI3 is shown in white columns, even staining throughout the cytosol and nucleus is shown in gray columns and black columns represent cells with predominantly cytosolic fluorescence. Data shown represent mean ± s.d. scored per group from 3 independent experiments of 100 cells each. (2.17 MB TIF) [file pone.0007471.s004.tif]
