# Supplementary material for: Point Mutations in GLI3 Lead to Misregulation of its Subcellular Localization
Source: PLoS One. 2009 Oct 15;4(10):e7471. doi: 10.1371/journal.pone.0007471 (PMC2758996; doi:10.1371/journal.pone.0007471)
Supplement: Table S1 — a: Sequences of siRNA's used in this study. Table S1b: Sequences of primers used for real-time PCR experiments. (0.05 MB DOC) [file pone.0007471.s005.doc]

Table S1a:

| Oligo-Nr. | Target gene | Target Sequence (5’-3’) |
| --- | --- | --- |
| 3 | α4 | CAAGAGAGGCATCAACTTCTA |
| 4 | α4 | CTCGTTGCTATGGCATCTCAA |
| - | non silencing | AATTCTCCGAACGTGTCACGT |
| 8 | MID1 | TTGAGTGAGCGCTATGACAAA |
| 9 | MID1 | AAGGTGATGAGGCTTCGCAAA |
| 10 | MID1 | TAGAACGTGATGAGTCATCAT |

Table S1b:

| Primer-name (realtime-PCR) | Target gene | Sequence (5’-3’) |
| --- | --- | --- |
| GAPDH-f1 | GAPDH | CCACCCATGGCAAATTCC |
| GAPDH-r1 | GAPDH | TGGGATTTCCATTGATGACAAG |
| GAPDH-f2 | GAPDH | CCACATCGCTCAGACACCAT |
| GAPDH-r2 | GAPDH | AAATCCGTTGACTCCGACCTT |
| CCND1-f1 | CCND1 | TGGGTCTGTGCATTTCTGGTT |
| CCND1-r1 | CCND1 | GCTGGAAACATGCCGGTTAC |
| CCND1-f2 | CCND1 | TGCGCTGCTACCGTTGACT |
| CCND1-r2 | CCND1 | AGCGATTGTAATATTTCCAAACC |
| MID1-f1 | MID1 | CTGCCAGGTCTGGTGTCATG |
| MID1-r1 | MID1 | AATCAGGCTTAGGGCCCTTCT |
| MID1-f2 | MID1 | TTGGAATGGTCCATGAATTAAGG |
| MID1-r2 | MID1 | CAAACTAGAACCAATGCCAGAGTTC |
| α4-f1 | α4 | CCCTCACCATGAAACAAGTCAA |
| α4-r1 | α4 | GCCCGCTGCAAATGATCT |
| α4-f2 | α4 | GGCTATGGGAACCGACAGAA |
| α4-r2 | α4 | CTGCAGTCCTGTGGTGTTGTG |
