# Supplementary material for: Point Mutations in GLI3 Lead to Misregulation of its Subcellular Localization
Source: PLoS One. 2009 Oct 15;4(10):e7471. doi: 10.1371/journal.pone.0007471 (PMC2758996; doi:10.1371/journal.pone.0007471)
Supplement: Table S2 — Summary of the subcellular localization of GFP-GLI3 (N-terminally tagged) and GLI3-GFP (C-terminally tagged) after knock-down of alpha4 using different siRNAs. Visualization and scoring were performed exactly as described in the legend to Fig. 1. (0.04 MB DOC) [file pone.0007471.s006.doc]

Table S2

| Cell line | Transfected with... | Subcellular localization of GLI3 (number of cells in %; average ± s.d.) | | |
| --- | --- | --- | --- | --- |
|  |  | nucleus | nucleus & cytosol | cytosol |
| HeLa | GLI3-GFP | 53.0 ± 8 | 38.0 ± 8 | 9.0 ± 5 |
| HeLa | GFP-GLI3 | 51.0 ± 3 | 36.3 ± 2 | 12.7 ± 3 |
| HeLa | GFP-GLI3 + control siRNA | 50.3 ± 5 | 38.0 ± 2 | 11.7 ± 3 |
| HeLa | GFP-GLI3 + α4-siRNA-4 | 30.7 ± 5 | 55.3 ± 4 | 14.0 ± 9 |
| HeLa | GFP-GLI3 + α4-siRNA-3 | 22.0 ± 3 | 56.7 ± 5 | 21.3 ± 3 |
| HeLa | GLI3-GFP + control siRNA | 52.0 ± 4 | 34.0 ± 9 | 14.0 ± 9 |
| HeLa | GLI3-GFP + α4-siRNA-3 | 35.3 ± 5 | 51.3 ± 8 | 13.3 ± 6 |
| HeLa | GLI3-GFP + α4-siRNA-4 | 36.0 ± 7 | 51.3 ± 3 | 12.7 ± 10 |
